# ATG9B regulates mitochondrial integrity and apoptotic tumor cell death

**DOI:** 10.1091/mbc.E25-07-0334

**Published:** 2026-04-10

**Authors:** Hong Cao, Lixia Guo, Jing Chen, Eugene Krueger, Gina Razidlo, Mark A. McNiven

**Affiliations:** ^a^Department of Biochemistry and Molecular Biology, Mayo Clinic, Rochester, Minnesota 55905; ^b^Division of Gastroenterology & Hepatology, Mayo Clinic, Rochester, Minnesota 55905; Nanyang Technological University

## Abstract

It is well established that many tumor types possess defective autophagic pathways. Several studies have reported that the transmembrane, autophagic lipid scramblase ATG9B is altered in multiple cancers, suggesting that this dysregulation could contribute to oncogenesis. Therefore, the goal of this study was to define the cellular distribution of ATG9B in two different tumor cell types and to provide insights into its cellular function. Surprisingly, we found that ATG9B shows a modest association with autophagic structures and exhibits a unique and prominent localization to mitochondria, in contrast to its related form ATG9A. Upon expression of tagged ATG9B forms, this mitochondrial distribution was accompanied by aberrant changes in mitochondrial morphology as well as a reduction in the mitochondrial membrane potential and the release of mtDNA. Few indicators for ATG9B-dependent mitophagy were noted. Instead, ATG9B overexpression led to pronounced apoptotic cell death as assessed by a variety of indicators. Further, we find that the N-terminal sequence of ATG9B acts as a mitochondrial targeting domain and that expression of this peptide alone can induce apoptotic cell death. These findings provide new insights into a putative cellular localization and function for ATG9B.

## Abstract (executive summary)

- The transmembrane protein ATG9B has been implicated in contributing to the autophagic process in mammalian epithelium.
- This current study demonstrates that in tumor cells of the lung and liver, ATG9B expression supports a modest autophagic response.
- More pronounced is its association with the mitochondrial surface via an N-terminal leader sequence where it disrupts mitochondrial structure and induces apoptotic cell death.
- This study demonstrates a new location and function for this transmembrane protein.

## INTRODUCTION

Autophagic pathways have been shown to play a role as tumor suppressors in the early stages of carcinogenesis by removing damaged organelles that can potentiate the generation of toxic proteins, DNA damage, reactive oxygen species (ROS), and inflammation (for reviews, refer to Assi and Kimmelman, 2023; Debnath *et al.*, 2023). A collection of studies has found that an inactivation of key autophagic genes leads to neoplasia. For example, Beclin1 was among the first autophagy genes to be linked to tumorigenesis. Monoallelic deletion of Beclin1 occurs in 40%–75% of cases of human sporadic breast, ovarian, and prostate cancers (Liang *et al.*, 1999; Qu *et al.*, 2003). Further, gene expression analysis in breast invasive ductal carcinomas (IDCs) have revealed significantly lower mRNA expression of ATG2B, ATG4D, ATG9A, and ATG9B compared with matched normal tissues (Zhang *et al.*, 2016).

As an extension of these findings, we probed human lung and liver cancer cell lines by Western blot analysis with a library of different autophagic protein antibodies to identify members of this pathway showing modest expression, which could result in unchecked cell growth. From this screen, we identified ATG9B as an autophagic protein that was modestly expressed in the cell lines examined. The ATG9 family is expressed as a single protein in yeast but as two distinct forms in humans, ATG9A and ATG9B. These members have four transmembrane and two intramembrane domains and may represent the only transmembrane proteins of the core autophagic machinery (Chang *et al.*, 2021). Most studied has been the ATG9A paralogue that has been shown by others to associate with vesicular tubular clusters, where it is believed to provide a supply of membrane lipids to the nascent phagophore (Judith and Tooze, 2019), while also aiding in the closure of more mature autophagosomes (Javed *et al.*, 2025). Consistent with this latter function is the participation of this protein in plasma membrane repair (Claude-Taupin *et al.*, 2021). The enzymatic activity of ATG9A appears to play a role in lipid mobilization either from membranes (Mailler *et al.*, 2021) or lipid droplets (Mailler *et al.*, 2021) via a scramblase activity that flips phospholipids between membrane leaflets (Maeda *et al.*, 2020)

In comparison to ATG9A, substantially less is known about ATG9B. While sharing approximately 40% identity with ATG9A, the greatest conservation resides within the lipid-binding and lipid docking sites. Unique domains are found at the N- and C-termini, suggesting these may contribute to a distinct distribution and function. One alteration is the absence of the autophagy-relevant ULK1 phosphorylation site at Ser14 found only in ATG9A (Zhou *et al.*, 2017). Despite these sequence disparities, a recent study has demonstrated that ATG9B does indeed possess lipid scramblase activity that is capable of substituting for ATG9A to support the autophagic process by delivering lipid to the nascent phagophore (Judith and Tooze, 2019). Whether any potential tumor suppressive activity of ATG9B is based upon an autophagic role is somewhat unclear. For example, experimental reduction of ATG9B in hepatocellular carcinoma cells decreases autophagic potential, leading to an increase in ubiquitylated proteins and ER-stress-based apoptotic cell death (Wang *et al.*, 2017). Further, while ATG9B appears to be protective in head and neck cancers, the authors concluded that this is likely to be via a noncanonical, autophagy-independent process, suggesting that ATG9B may have additional tumor suppressive functions (Guo *et al.*, 2022).

In this study, our goal was to define the intracellular distribution of ATG9B and its role as a potential tumor suppressor in lung and liver cancer cell lines that display an exceptionally low expression of this protein. As reported by others (Mattera *et al.*, 2017; Judith and Tooze, 2019), we find endogenous or expressed tagged forms of ATG9B localize to internal secretory and endocytic membranes such as the Golgi apparatus and endosomes. Of marked interest to us was the unreported yet prominent localization to mitochondria, a distribution not shared with the ATG9A form. Importantly, this mitochondrial distribution was lost upon removal of the N-terminal 104 amino acid domain that appeared to act as a targeting sequence, as this domain alone was able to localize to mitochondria. Further, expression of full-length ATG9B induced pronounced changes in mitochondrial shape, cristae organization, membrane potential, and genome stability.

Given the role of the ATG9 proteins in autophagy and the mitochondrial localization of ATG9B, we investigated ATG9B as a putative mitophagy protein, with the expectation of seeing an increase in autophagic or mitophagic responses induced through the expression of high levels of ATG9B. This exogenous expression did induce a general autophagic response as measured by changes in LC3 lipidation. However, a mitophagic response was minimal as assessed by a flow cytometry-based mtKeima assay. Instead, ATG9B expression led to substantial apoptotic cell death as reflected by a marked increase in cleaved caspase-3, Annexin V binding to the plasma membrane, nuclear deformation, and BAX activation. Most graphic was the rapid association of ATG9B to mitochondria before cell death as viewed by live cell imaging. Taken together, these findings suggest that while able to support a general autophagic response, ATG9B is a mitochondrial-associated protein that may also play a central role in apoptotic cell death.

## RESULTS

### ATG9B is targeted to tumor cell mitochondria via a unique N-terminal sequence

**FIGURE 1:**
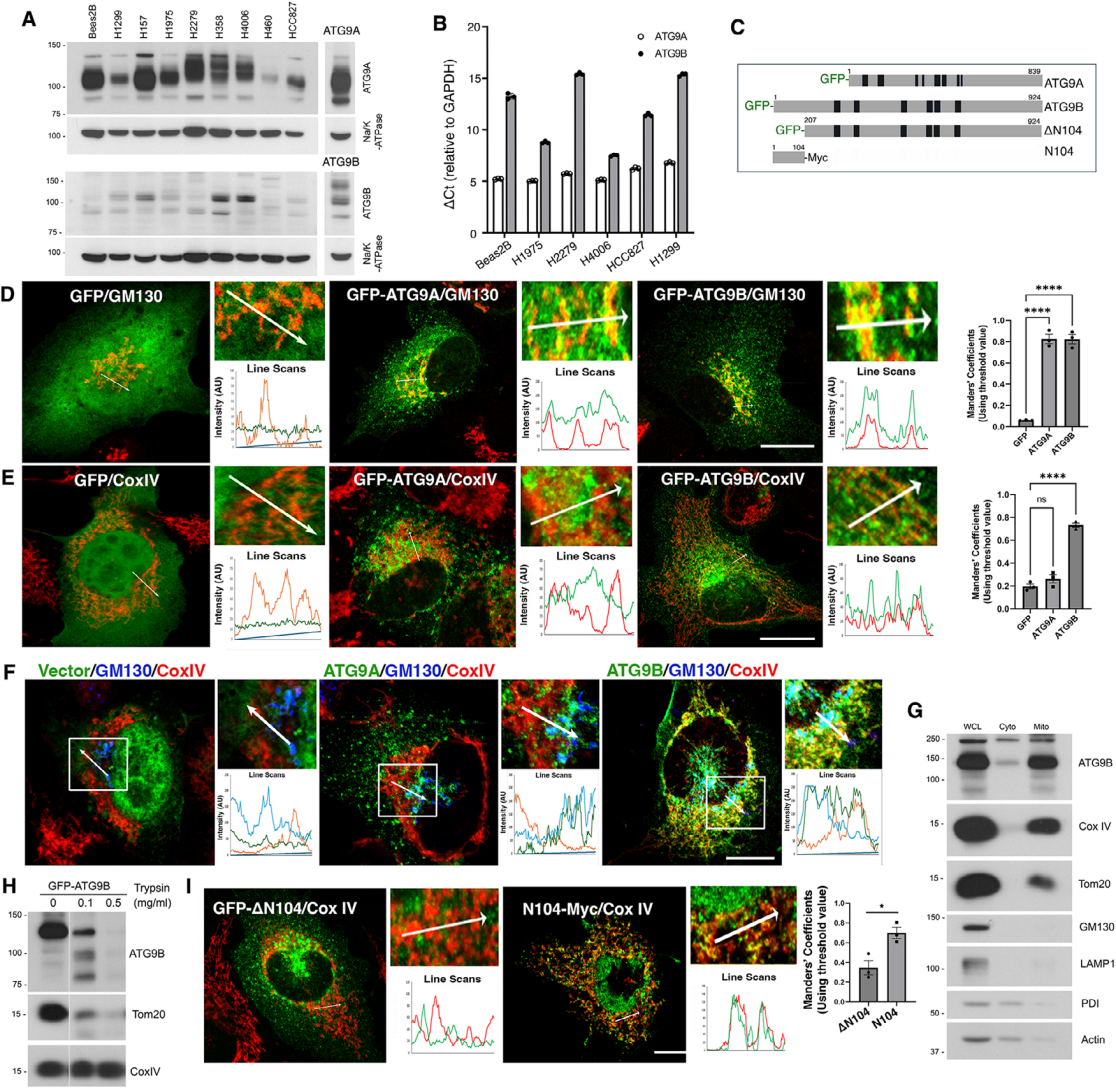
ATG9B is directed to the mitochondria via an N-terminal domain. (A) Western blot analysis showing endogenous expression levels of ATG9A and ATG9B using membrane protein fractions from eight different lung cancer cell lines and a non-cancerous cell line (Beas2B). H1299 cells were transfected with a cDNA encoding ATG9A or ATG9B as a positive control (right). (B) Quantitative real-time PCR (qRT-PCR) analysis of ATG9A and ATG9B mRNA expression in the indicated cell lines. Data are presented as ΔCt values normalized to GAPDH. (C) Schematic representation of constructs used in this study: GFP-tagged ATG9A, full-length and truncated forms of ATG9B (GFP-ATG9B and GFP-ΔN104), and a Myc-tagged peptide consisting of the first 104 amino acids of the N-terminus (N104-Myc). Predicted transmembrane domains are depicted in black with cytosolic and luminal segments shown in gray. (D–F) Confocal images, corresponding line scans, and Manders’ Coefficients graphs comparing the distribution of GFP vector, GFP-ATG9A, or GFP-ATG9B with Golgi, mitochondria, or both. Enlargements and corresponding line scans along the arrows are shown in the right column of each image depicting the colocalization. H1299 cells overexpressing GFP vector, GFP-ATG9A, or GFP-ATG9B co-stained for (D) the Golgi marker GM130 (red), (E) for the mitochondria marker Cox IV (red), (F) for GM130 (blue) and Cox IV (red). (G) Western blot analysis of whole cell lysate (WCL), cytosolic (Cyto) and mitochondrial (Mito) fractions of H1299 cells transfected with GFP-ATG9B showing the marked enrichment of ATG9B in the mitochondrial fraction with minimal contamination from Golgi (GM130), lysosome (LAMP1), ER (PDI) and cytosolic (Actin) markers. (H) Western blot analysis of trypsin-treated mitochondria isolated from GFP-ATG9B-transfected H1299 cells. The protease reduces levels of ATG9B and TOM20 but not the inner membrane Cox IV. (I) Distribution of GFP-ΔN104 shows a reduced colocalization with Cox IV (red), whereas N104-Myc (green) alone shows an association with mitochondria (Cox IV, red). Manders’ coefficients quantifying the colocalization between GFP-ΔN104 and N104-Myc with mitochondria. Graphs of Manders’ coefficients shown in D, E, and I each represent the mean ± SEM of three independent biological replicates (26–38 cells in total per condition). Statistical analyses were performed using an unpaired two-tailed Student's *t* test. *p < 0.05, ***p < 0.001. Scale bar, 10 µm.

To evaluate the endogenous expression of ATG9B in comparison with the more well-studied ATG9A protein across a variety of different cancer cell lines, we performed Western blotting using enriched membrane protein fractions. Consistent with RNAseq data from the Human Protein Atlas, ATG9A was detected at substantial levels in all eight lung cancer cell lines tested (H1299, H157, H1975, HCC2279, H358, HCC4006, H460, and HCC827) as well as an immortalized, non-neoplastic, lung bronchiole cell line (Beas2B). In contrast, ATG9B expression was markedly low, if not absent, across all the cell lines examined (Figure 1A). Transfection with a cDNA expressing ATG9A or ATG9B served as a positive control. Multiple Western blot bands were detected for both ATG9A and ATG9B proteins, suggesting post-translational modifications. Consistent with the protein results of others, qRT-PCR analysis confirmed the high ATG9A and low ATG9B mRNA abundance, with ATG9B exhibiting higher ΔCt values than ATG9A in each cell line examined (Figure 1B). This corresponds to approximately five to 1000-fold higher levels of the ATG9A form compared with ATG9B within each cell line analyzed. The non-neoplastic cell line Beas2B exhibits ATG9B levels comparable to those of the tumor cells tested.

Currently, the subcellular distribution of ATG9B remains poorly defined. While most of this study was conducted on the human lung carcinoma cell line H1299, key findings were confirmed in another human lung carcinoma cell line (HCC827) and a human hepatoma cell line (Hep3B). To define and compare the subcellular localization of ATG9A and ATG9B, GFP-tagged constructs of ATG9A or ATG9B were expressed in both lung and liver tumor cells (Figure 1C). Following fixation and co-staining with organelle markers, both GFP-ATG9A and GFP-ATG9B were found to localize similarly with the Golgi marker GM130, as well as in the peripheral cytoplasm (Figure 1D). Surprisingly, GFP-ATG9B was also found to colocalize predominantly with the mitochondrial marker Cox IV (Figure 1E), a pattern not observed for GFP-ATG9A (Figure 1E). These colocalization patterns were quantified using either line scans or Manders’ coefficients (Figure 1, D and E). ATG9B is also expressed in the tumor cell line Hep3B, where it associates with mitochondria (Supplemental Figure S1, B and E) and the Golgi (Supplemental Figure S1E). Further, super-resolution imaging of these cells for ATG9B indicated an association with the outer mitochondrial membrane (TOM20), the organelle interior labeled with Cox IV (Supplemental Figure S1F).

As staining of GFP-ATG9B with either GM130 or Cox IV indicates that ATG9B may associate with distinct organelles, triple fluorescence imaging for GM130, Cox IV, and GFP-ATG9B was performed. These images indicate that ATG9B localizes to both the Golgi and mitochondria within the same cell, with the corresponding line-scan revealing a prominent peak of GFP-ATG9B signal that coincides with Cox IV and a reduced peak with GM130 (Figure 1F). In contrast, GFP-ATG9A colocalizes largely with the Golgi but not with Cox IV, as indicated by the single peak overlapping with GM130 (Figure 1F).

The association of ATG9B with mitochondria was tested further by Western blot analysis of subcellular organelles isolated from H1299 cells. As depicted in Figure 1G, ATG9B is prominent in the mitochondrial fraction, which exhibits absent or minimal contamination from other organelles, as indicated by the lack of detectable markers for other organelle fractions, including GM130 (Golgi), LAMP1 (lysosome), and PDI (ER). As a biochemical approach to further support the high-resolution imaging of ATG9B association with the outer mitochondrial membrane, as depicted in Supplemental Figure S1F, we performed a protease protection assay. Mitochondria were isolated from H1299 cells and incubated with increasing concentrations of trypsin for 20 min before Western blot analysis. Consistent with an outer membrane localization, levels of intact ATG9B were reduced by increasing concentrations of trypsin, as was the outer membrane protein TOM20 (Figure 1H). In contrast, levels of the inner membrane protein Cox IV remained constant, indicating protection from the protease.

To identify the factors influencing the differential subcellular distributions between the two ATG9 paralogues, the protein sequences were compared and analyzed. The ATG9 domain and transmembrane regions appear highly conserved, with the greatest differences found at the N- and C-termini. Most conspicuous was a 143 amino acid extension from the N-terminus of ATG9B that includes a proline-rich region, and some portions enriched with polar residues. We hypothesized that this extended N-terminal sequence of ATG9B might contribute to its mitochondrial localization. To test this premise, a truncated form of ATG9B was constructed in which the first 104 amino acids were deleted, termed GFP-ATG9BΔN104 (Figure 1C). Expression of this truncated protein in H1299 cells revealed that the mitochondrial association of this ATG9B form was reduced (Figure 1I). To pursue this finding further, a second construct containing only the first 104 amino acids of ATG9B and including a C-terminal Myc tag, termed N104-Myc (Figure 1C), was tested for co-localization with Cox IV and showed a strong mitochondrial distribution (Figure 1I). The importance of this ATG9B mitochondrial-targeting, N-terminal domain was confirmed in hepatoma cells (Hep3B) that possess more clearly defined mitochondria and display a near exact co-distribution (Supplemental Figure S1, B and D), while an ATG9B protein missing the N-terminal domain (GFP-ATG9BΔN104) shows minimal mitochondrial association (Supplemental Figure S1C). Together, these results indicate a strong correlation between the N-terminal sequence of ATG9B and its mitochondrial localization.

### Alterations in mitochondrial structure and function by ATG9B expression

**FIGURE 2:**
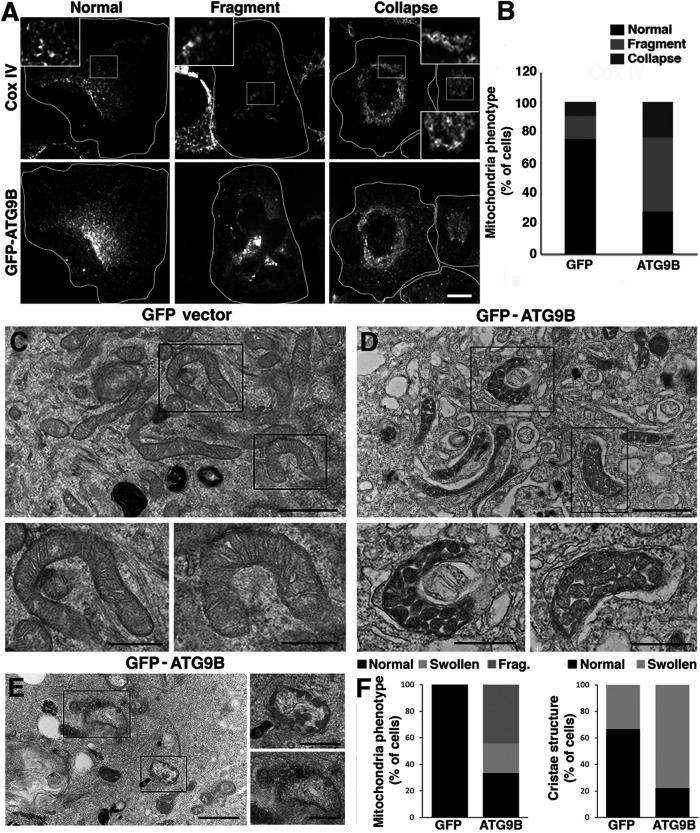
Overexpression of ATG9B disrupts mitochondrial morphology in H1299 cells. (A) Confocal images depicting H1299 cells stained for Cox IV and expressing GFP-ATG9B, illustrating three mitochondrial subtypes: normal, fragmented, and collapsed. Normal-looking mitochondria are characterized by well-defined, elongated tubular structures. Fragmented mitochondria display small, rounded, or punctate structures with noticeably reduced length compared with normal mitochondria and often with reduced Cox IV staining. Collapsed mitochondria exhibit perinuclear localization and diminished peripheral distribution. (B) Bar graph representing the distribution of the three observed subtypes of GFP-ATG9B expressing cells as compared with GFP vector expressing cells (∼200 cells per group per experiment, *n* = 3). (C–E) Representative TEM images of H1299 cells transfected with control GFP vector (C), or GFP-ATG9B (D and E) for 27 h. Enlarged views of the boxed regions are shown in the bottom panels to illustrate the categorized subtypes. (C) Representative mitochondria in control cells display elongated tubular structures with well-defined, densely packed, and flat cristae. (D and E) Mitochondria in GFP-ATG9B-transfected cells display structural alterations, including swollen cristae, with round or irregular shapes, and increased cristae volume. (F) Statistical summation of the observed mitochondrial ultrastructural alterations as described in (C–E). Mitochondria were categorized using two complementary criteria: (i) overall mitochondrial morphology, classified as normal, fragmented, or swollen (left panel); and (ii) cristae architecture, classified as normal or swollen (right panel). Six GFP vector-expressing cells and nine GFP-ATG9B-expressing cells were counted. Histogram graphs show the percentage of cells exhibiting each mitochondrial phenotype. Scale bars: (A) 10 µm; (C–E) 1 µm (widefield view), 0.5 µm (enlarged view).

To test if ATG9B expression might exert an effect on mitochondrial shape and integrity, transfected tumor cells were examined with both confocal and transmission electron microscopy. We found that 27 h post-transfection, mitochondria in H1299 cells displayed a variety of altered morphologies that were categorized into three subtypes and included elongated tubular structures characteristic of normal mitochondria; punctate, rounded, structures that appeared fragmented; and finally, a condensed perinuclear localization with diminished peripheral distribution, suggesting a collapse of the mitochondrial network and a loss of structural integrity (Figure 2A). As shown in Figure 2B, around 80% of the GFP vector-transfected control cells exhibit elongated tubular mitochondria. In contrast, approximately 80% of the ATG9B-overexpressing cells display mitochondria with either fragmented or collapsed morphologies. Subsequently, cells transfected with the GFP vector or GFP-ATG9B were fixed on gridded coverslips that aided in cell identification analysis. As shown in Figure 2, C and D, ultrastructural analysis supported the confocal imaging and showed ATG9B expression inducing alterations in mitochondrial shape and size. Further, these images revealed significant changes in the inner cristae that became swollen or vesiculated compared with control cells. In many instances, GFP-ATG9B-overexpressing cells displayed mitochondria with an almost complete loss of cristae and appeared empty or hollow (Figure 2E; Supplemental Figure S2, F and G).

To quantify these ultrastructural changes, control and transfected cells were classified into three categories based on the morphology of their mitochondria. These include mitochondria that appeared as normal and elongated with densely packed, flat cristae, round with irregular cristae and enlarged cristae volume, or finally, swollen or missing cristae. As shown in Figure 2F, most mitochondria in control GFP vector-transfected cells exhibit normal morphology, with cells containing mitochondria with normal features, and 33% of cells displaying mitochondria with swollen cristae. In contrast, ATG9B-overexpressing cells exhibit a loss of normal features, with over 40% of cells containing fragmented mitochondria and almost 80% of cells showing mitochondria of the swollen cristae subtype (Figure 2F). The effects of ATG9B expression on mitochondrial shape and integrity were also assessed by light microscopy and TEM on a second lung cancer cell line, HCC827 (Supplemental Figure S2, A–H). Similar structural disruptions of mitochondria were observed in these cells due to elevated levels of ATG9B.

**FIGURE 3:**
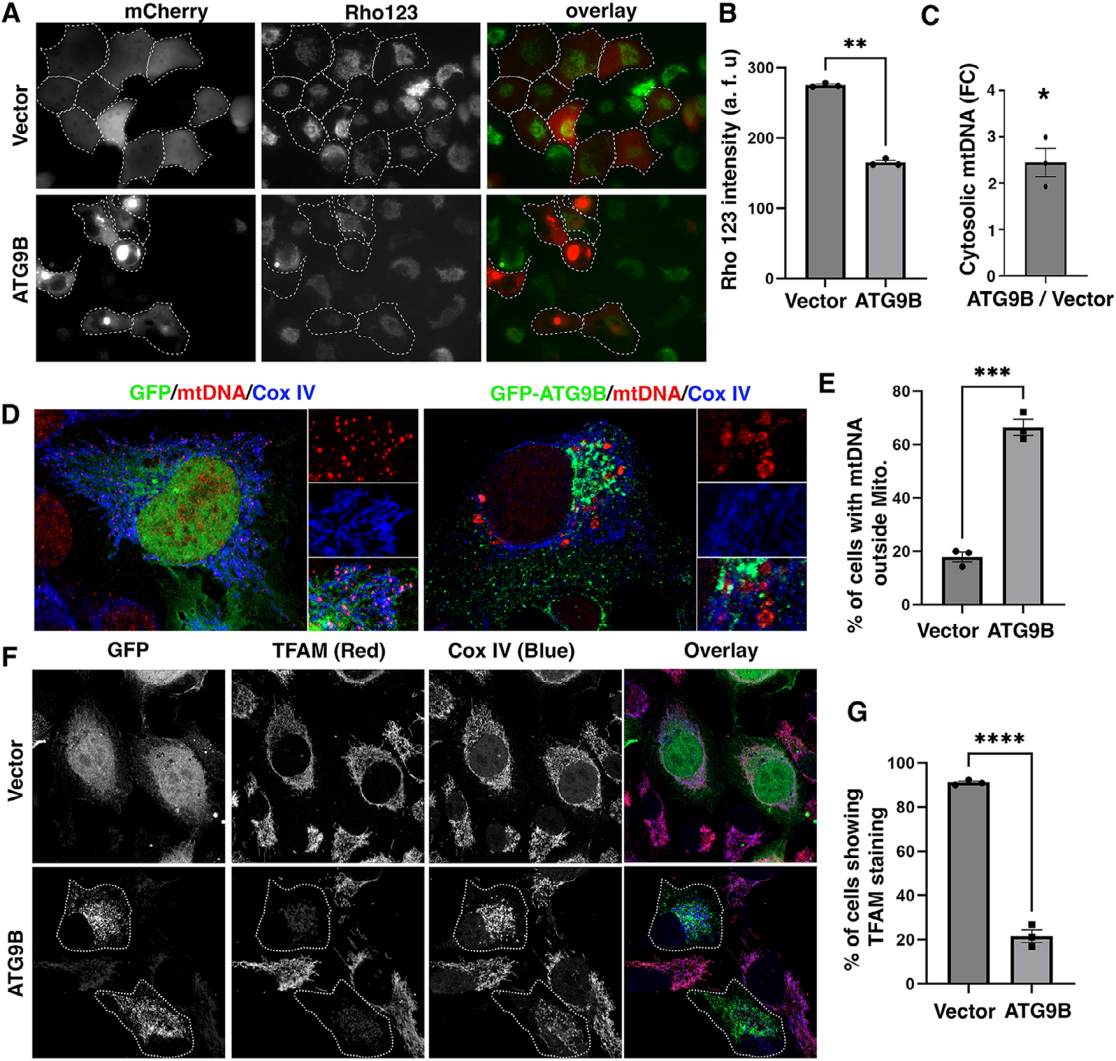
Overexpression of ATG9B disrupts the membrane potential and genomic stability of mitochondria. (A) Representative images of H1299 cells transfected to express either mCherry vector control (Vector) or mCherry-ATG9B (ATG9B) and incubated with Rho123, as an indicator of membrane potential (green). A reduced number and intensity of Rho123 staining in cells overexpressing mCherry-ATG9B is observed compared with control cells. (B) Bar graph depicting the mean fluorescence intensity of Rho123 for mCherry vector or mCherry-ATG9B expressing cells, based on three independent experiments (>50 cells per group per experiment, *n* = 3). a.f.u: arbitrary fluorescence units. (C) Cytosolic mitochondrial DNA (mtDNA) levels were quantified by qPCR in cells following expression of empty vector or ATG9B for 48 h. These levels were normalized to the corresponding whole-cell mtDNA and expressed as fold change (FC) relative to control, which was set to 1 for each biological replicate (*n* = 3). (D) Confocal images of cells transfected with either GFP vector or GFP-ATG9B and stained with antibodies against DNA (red) and mitochondrial Cox IV (blue). GFP vector expressing cells show small mtDNA nucleoids overlapping with mitochondria, while a vast majority of cells overexpressing GFP-ATG9B display enlarged mtDNA aggregates that appear dislocated from mitochondria (D). (E) Bar graph illustrating the percentage of cells with displaced DNA (∼30 cells per group per experiment, *n* = 3). (F) Confocal images of the mitochondrial transcription factor TFAM (red) and Cox IV (blue) showing a substantial loss of TFAM staining in GFP-ATG9B expressing cells compared with those expressing the GFP vector control. (G) Bar graph illustrating the percentage of cells with detectable TFAM staining (∼100 cells per group per experiment, *n* = 3). All bar graphs represent mean ± SEM. Significance was determined using a two-tailed Student's *t* test for data in (B, E, and G). Fold change data in (C) were analyzed using a one-sample *t* test against a theoretical value of 1. *p < 0.05, **p < 0.01, ***p < 0.001.

Based on the significant disruption of normal mitochondrial structure by ATG9B overexpression in cells, we predicted that these organelles were likely functionally compromised. Such damage could be reflected by a loss of mitochondrial membrane potential (MMP). As the preservation of MMP depends on mitochondrial integrity, a vital fluorescent dye, Rhodamine 123 (Rho123) was used, as its selective accumulation within mitochondria is dependent upon an intact electrochemical gradient. As anticipated, cells transfected to overexpress GFP-ATG9B exhibited reduced incorporation and retention of Rho123 staining compared with control cells, indicating that ATG9B overexpression compromises mitochondrial membrane potential, consistent with the observed mitochondrial structural damage (Figure 3, A and B).

An additional criterion for mitochondrial integrity is the capacity to retain their mtDNA organized into nucleoids with associated structural transcription factors such as TFAM, which is crucial for maintaining mtDNA transcription, replication, and stability. Mitochondria are known to expel both mtDNA and TFAM when damaged or structurally compromised. To assess whether ATG9B-induced mitochondrial damage leads to increased mtDNA leakage, we performed quantitative PCR (qPCR) to measure the cytosolic mitochondrial DNA (mtDNA) level following cell fractionation. As displayed in Figure 3C, cytosolic mtDNA levels in GFP-ATG9B-transfected cells were approximately 2.5-fold higher than those in GFP control cells. In addition, immunofluorescence staining revealed mtDNA nucleoid morphological changes that are typically associated with mitochondrial damage. In control GFP vector expressing H1299 cells, mitochondrial DNA (mtDNA) appears as small nucleoids colocalized with Cox IV, reflecting a normal mitochondrial state (Figure 3D). In contrast, GFP-ATG9B overexpressing cells show mtDNA clumped into large aggregates both within and outside the mitochondrial margins (Figure 3D). Over 60% of ATG9B-overexpressing cells have cytosolic mtDNA, compared with less than 20% in control cells (Figure 3E). Further, the mitochondrial localization of TFAM is substantially reduced in ATG9B-overexpressing cells (Figure 3, F and G). These changes appear consistent with structurally compromised mitochondria.

### Induction of apoptosis by ATG9B expression

As ATG9B has been implicated in several autophagic processes (Wang *et al.*, 2017; Chiduza *et al.*, 2024) and appears to associate with mitochondria as described above, we hypothesized that it may induce a mitophagic response in overexpressing cells that is attenuated in tumor cells exhibiting low expression levels. To test this premise, Western blotting was used to assess the protein levels of Cox IV and TOM20 in control versus ATG9B-expressing cells. However, no statistically significant differences in these protein levels were observed between GFP vector and ATG9B-transfected cells (Supplemental Figure S3, A–C). In contrast, the level of LC3B-II, a nonselective autophagy marker, is significantly increased (Supplemental Figure S3D), suggesting that ATG9B overexpression may influence general macroautophagy without specifically inducing mitophagy. To further explore whether ATG9B overexpression influences mitophagy, the mtKeima assay was implemented to monitor mitophagy via flow cytometry. This is a pH-sensitive ratiometric fluorescent protein that associates with mitochondria and undergoes a spectral shift to a longer (red) wavelength upon entry into the acidic lysosome as a reflection of mitophagic activity, which can be measured via flow cytometry (Wang, 2020). As shown in Supplemental Figure S3, E and F, treatment of mtKeima-transfected H1299 cells with the mitophagy-inducing oxidative phosphorylation uncoupler, carbonyl cyanide 3-chlorophenolhydrazone (CCCP), increases the number of mitophagic cells from 5% to 15% in control GFP-transfected cells. Lastly, as the key mitophagic E3 ligase PARKIN is absent from all but one of the lung tumor cell lines studied here, we attempted to favor mitophagy by expressing both ATG9B and PARKIN together. As shown in Figure S3G, CCCP treatment led to an expected accumulation of PINK1 and phosphorylation of ubiquitin at Ser65 in the presence of PARKIN, indicating the activation of the PINK1/PARKIN mitophagy pathway. However, the levels of the mitophagy receptors NIX and BNIP3, as well as the mitochondrial markers TOM20 and Cox IV, remained comparable between GFP vector and GFP-ATG9B-transfected cells under both basal and CCCP treatment conditions (Supplemental Figure S3, G–K), suggesting that ATG9B overexpression does not enhance mitophagy under both conditions.

**FIGURE 4:**
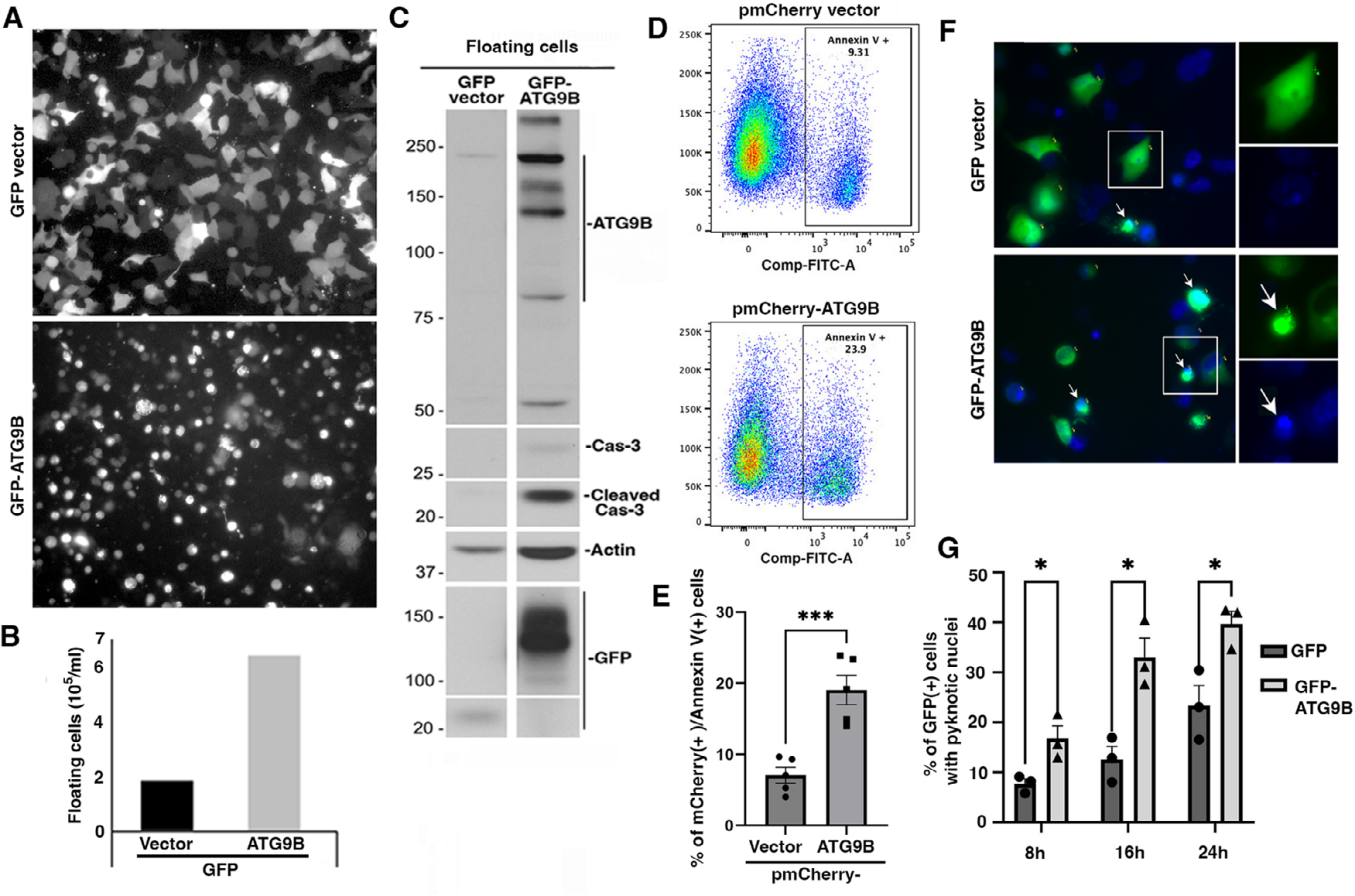
ATG9B expression promotes tumor cell apoptosis. (A) Representative low magnification images of H1299 cells transfected with GFP vector or GFP-ATG9B for 36 h. Expression of ATG9B induces cells to round up and release from the substrate. (B) Quantification of the number of floating cells resulting from ATG9B expression in a single experiment. (C) Floating cells were collected and subjected to Western blot analysis, which confirmed the cleavage of caspase-3 and ATG9B expression in the floating cell fraction. (D) Representative flow cytometry analysis and (E) corresponding quantification of the percentage of Annexin V-positive cells in populations expressing either mCherry vector or mCherry-ATG9B. Cells were gated for mCherry-positive cells by gating before Annexin V analysis. Bars represent means ± SEM from five independent biological replicates (*n* = 5). (F) Nuclear Hoechst staining of cells transfected with either GFP vector or GFP-ATG9B for 8, 16, or 24 h. Arrows indicate pyknotic nuclei representative of apoptotic cells. Boxed regions provide higher magnification. (G) Bar graph representing the percentage of cells with pyknotic nuclei at the indicated times following transfection with GFP vector or GFP-ATG9B (∼150 cells per group per experiment counted). Bars represent means ± SEM (*n* = 3). Statistical analyses were performed using an unpaired two-tailed Student's *t* tests, *p < 0.05, ***p < 0.001.

A notable cellular response to ATG9B expression was the detachment of many transfected GFP-ATG9B-positive cells from the culture substrate compared with cells transfected with the GFP vector alone (Figure 4, A-C). To test if these floating cells had undergone apoptotic cell death, the floating cells were pelleted, lysed, and evaluated by Western blot for the apoptosis marker cleaved caspase-3. As depicted in Figure 4C, a substantial 13-fold increase in cleaved caspase was observed in the ATG9B-expressing cells compared with control cells 30 h post-transfection. As a second criterion, Annexin V binding to the cell surface via an interaction with externalized phosphatidylserine (PS) is a widely used assay that indicates the early stages of apoptosis. To this end, H1299 cells either expressing an mCherry vector or mCherry-ATG9B for 30 h were incubated with FITC-tagged Annexin V for 30 min before flow cytometry analysis. We observed that substantially more ATG9B-expressing cells were Annexin V positive compared with the vector-expressing control cells (Figure 4, D and E), indicative of an apoptotic response. Cells late in the apoptotic cascade exhibit marked changes in nuclear shape and integrity in a process termed nuclear pyknosis. To evaluate this response, vector or GFP-ATG9B-expressing H1299 cells were stained at various times post-transfection with the vital dye Hoechst 33342 (Figure 4, F and G). A substantial percentage of GFP-ATG9B-expressing cells displayed condensed and deformed nuclei compared with control cells.

**FIGURE 5:**
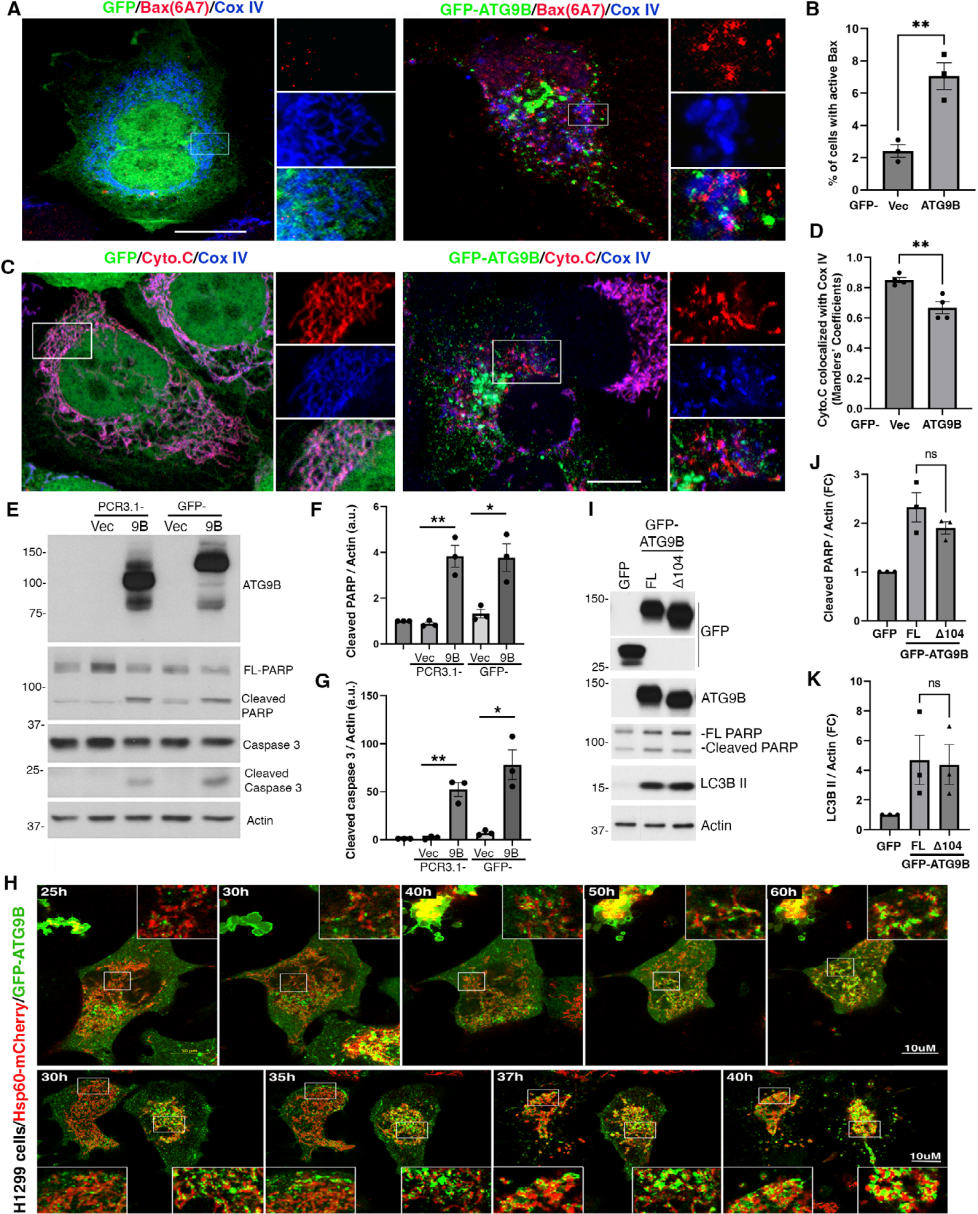
ATG9B expression triggers cell death through the mitochondrial apoptotic pathway. (A) Confocal images of H1299 cells transfected to express GFP or GFP-ATG9B for 24 h, then fixed and immunostained for active BAX (red) and Cox IV (blue). A substantial number of GFP-ATG9B expressing cells exhibit active BAX (6A7) staining compared with the GFP vector control cells, as represented in the adjacent graph (B; ∼80 cells per group per experiment, *n* = 3). (C) Cyto. C (red) and Cox IV (blue) staining of H1299 cells transfected to express GFP vector or GFP-ATG9B before fixation and staining. A redistribution of Cyto. C away from the mitochondria is observed in the ATG9B expressing cells compared with control cells, as represented in the accompanying bar graph (D; ∼15 cells per group per experiment, *n* = 4). (E) Western blot analysis of cleaved caspase-3 and cleaved PARP in H1299 cells or cells transfected with GFP-tagged or untagged ATG9B for 24 h. (F and G) densitometry quantification of cleaved PARP (F) and cleaved caspase-3 (Cas3; G) from three independent experiments showing a marked increase in the apoptotic indicators by ATG9B expression. (H) Images from two representative live-cell time-lapse confocal movies of H1299 cells transfected with GFP-ATG9B for 9 h before the start of image acquisition, then imaged for 40–60 h at 10-min intervals. The images reveal several distinct cells with increasing levels of GFP-ATG9B recruitment to the surface of mitochondria over time. Enlarged views of the framed regions highlight this increase. In some cells, this association reaches substantial levels, accompanied by cellular blebbing and death. Refer to Supplemental Video S2 and S3, corresponding to the top and bottom panels, respectively. Scale bar: 10 µm. (I) Western blot analysis of cleaved PARP and LC3B-II in H1299 cells transfected with GFP vector, GFP-ATG9B, or GFP-ATG9BΔN104. (J and K) densitometric quantification of cleaved PARP (J) and LC3B-II (K) from four independent experiments. Data in (B, D, F, and G) are represented as mean ± SEM; significance was determined by paired two-tailed Student's *t* tests. Data in (J and K) are normalized to the GFP control within each replicate; significance was determined by one-sample *t* tests against a theoretical mean of 1. ns: nonsignificant, *p < 0.05, **p < 0.01.

Finally, we aimed to define the central apoptotic pathway exhibited by GFP-ATG9B-expressing cells. Apoptosis can result from an alteration of the mitochondrial outer membrane permeability (intrinsic) mediated by the BCL-2 family member BAX (Oltval *et al.*, 1993). During the activation of the intrinsic apoptotic pathway, BAX is activated via a conformational change and subsequent translocation to the mitochondria, leading to membrane permeabilization and the release of cytochrome c. To investigate BAX activation and intrinsic apoptosis, control GFP vector or GFP-ATG9B expressing cells were fixed and co-stained for active BAX (6A7) and Cox IV. As shown in Figure 5, A and B, mitochondrial-associated active BAX was observed in three times as many of the GFP-ATG9B overexpressing cells compared with the GFP vector expressing control (Figure 5C). Consistent with this observation, cytochrome c showed decreased mitochondrial localization in the GFP-ATG9B expressing cells as quantified using Manders’ coefficients, consistent with cytochrome c release into the cytosol (Figure 5, C and D). Cytochrome c triggers the caspase cascade, leading to the cleavage of both caspase-3 and subsequently poly [ADP-ribose] polymerase (PARP; Chaitanya *et al.*, 2010). Indeed, cleaved caspase-3 and cleaved PARP were both elevated in GFP-ATG9B-transfected cells compared with GFP vector-expressing control cells as measured by Western blotting (Figure 5, E–G). Together, these responses are supportive of a canonical intrinsic apoptotic pathway activation due to ATG9B expression.

To better correlate the cellular effects of ATG9B expression with cellular dynamics and apoptosis, time-lapse confocal microscopy was performed on H1299 cells co-expressing GFP-ATG9B and Hsp60-mCherry as a mitochondrial marker. Image acquisition of live H1299 cells was performed 9 h post-transfection and continued for 40–60 h at 10-min intervals. As shown in the two representative fields in Figure 5H, and the accompanying videos (Supplemental Videos S2 and S3), ATG9B initially appears as small cytoplasmic puncta, some closely associated with tubular mitochondria (20–25 h post-transfection). Over time, ATG9B is markedly recruited to form distinct linear structures surrounding or aligning with mitochondria, leading to fragmentation and subsequently cellular blebbing and death. Taken together, these responses are supportive of a canonical intrinsic apoptotic pathway activation due to ATG9B expression.

From the data described above, mitochondrial recruitment of ATG9B appears to precede apoptotic cell death. To test if reducing ATG9B association with the mitochondria might attenuate this response, a truncation mutant ATG9BΔN104, missing the N-terminal 104 amino acids constituting the mitochondrial targeting sequence (shown in Figure 1I), was expressed in H1299 cells for 48 h before Western blot analysis of PARP. As shown in Figure 5I, ATG9BΔN104 induces PARP cleavage to a similar extent as full-length ATG9B, with no statistically significant difference between them (Figure 5J). Although the cleaved PARP densitometry signal for ΔN104 trends lower across the replicates, this difference does not reach statistical significance, indicating that ATG9B-induced cell death is not dependent solely on its N-terminal mitochondrial targeting sequence under the tested conditions. These data suggest that additional sequences or features of ATG9B beyond the first N-terminal 104 amino acids may contribute to its pro-apoptotic activity. Notably, ATG9BΔN104 also increases LC3B-II lipidation comparably to full-length ATG9B (Figure 5K), suggesting that ATG9BΔN104 retains autophagy-associated activity.

**FIGURE 6:**
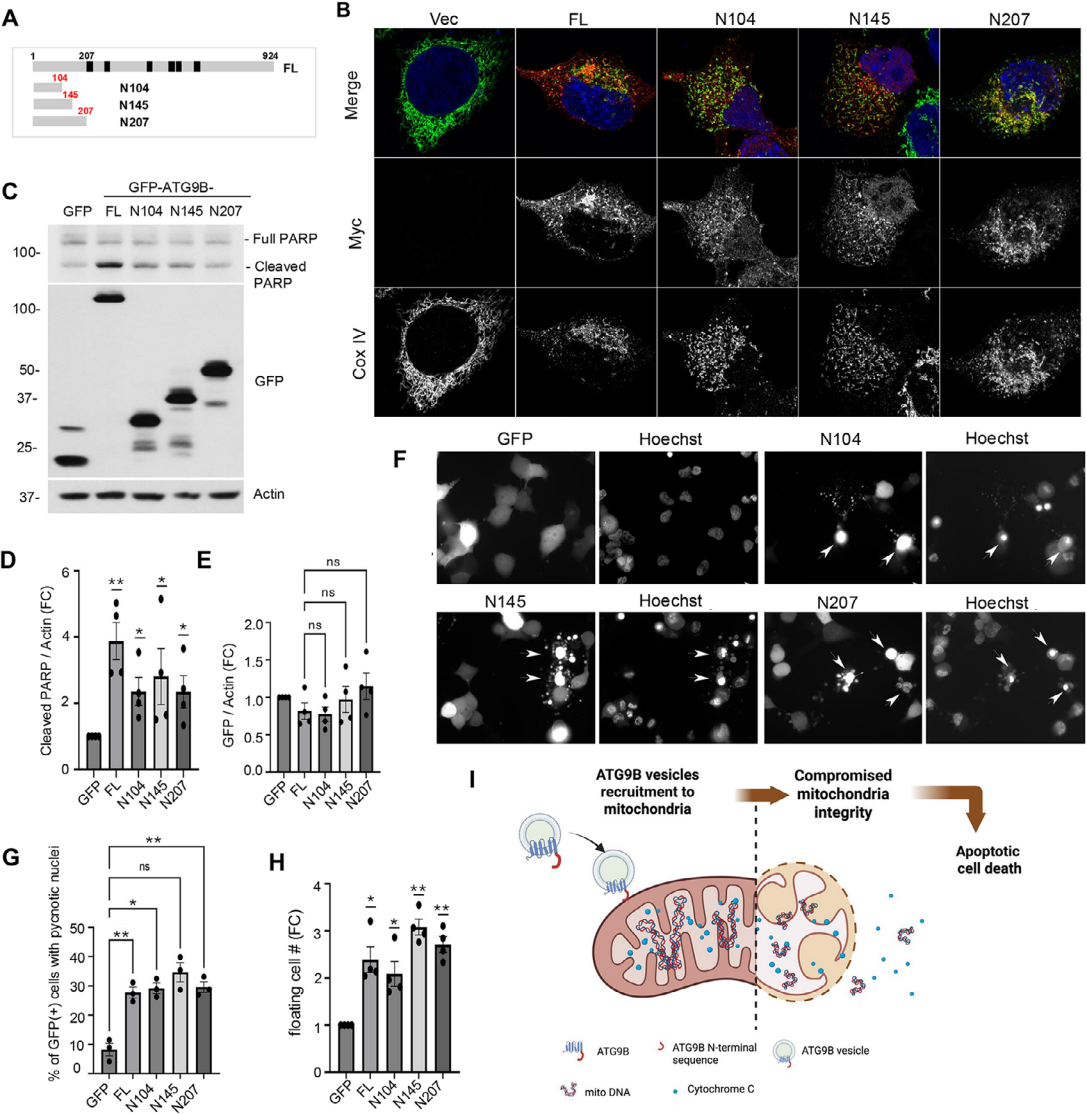
N-terminal peptides of ATG9B are sufficient to induce apoptosis and disrupt mitochondrial morphology independent of its scramblase activity. (A) Schematic representation of full-length ATG9B (FL) and N-terminal peptides comprising the first 104, 145, and 207 amino acids, designated as N104, N145, and N207, respectively. Predicted transmembrane or intramembrane domains are depicted in black, while cytosolic and luminal regions are shown in gray. (B) Representative confocal images of H1299 cells transfected with either the control pcDNA3.1 His/Myc vector or vectors expressing ATG9B full length (FL), N104, N145, or N207. Cells were immuno-stained for Cox IV (green) and Myc-tagged constructs (red). (C) Western blot analysis of cells transfected with GFP vector, GFP-tagged ATG9B full-length (FL), or GFP-tagged N-terminal peptide constructs. Densitometric quantification of cleaved PARP (D) and GFP (E) from *n* = 3 independent experiments corresponding to the immunoblot shown in (C). (F) Representative epifluorescence images of transfected cells showing GFP expression and nuclear staining with Hoechst. Arrowheads indicate pyknotic nuclei, characteristic of apoptotic cells. (G) Quantification of pyknotic nuclei among GFP-positive cells, expressed as the percentage of GFP-positive cells with pyknotic nuclei (∼50 cells per group per experiment, *n* = 3). (H) Quantification of the floating (non-adherent) cells for each transfection condition. (I) Schematic model illustrating how ATG9B, and its N-terminal domain, might mediate its translocation to mitochondria to induce organelle disruption and induction of apoptotic cell death. Bar graphs represent the mean ± SEM. Statistical analyses were performed using one-way ANOVA followed by multiple comparisons test (E, G), or one-sample *t* test for fold change analyses (D and H). ns: nonsignificant, *p < 0.05, **p < 0.01.

Finally, to test whether mitochondrial localization of ATG9B is sufficient to induce mitochondrial damage and cell death, N-terminal peptides comprising 104, 145, or 207 amino acids of ATG9B were expressed in H1299 cells. The N104 peptide includes the ATG9B mitochondrial targeting sequence, as shown in Figure 6A, while N145 extends beyond this region, representing the full sequence absent in ATG9A; N207 encompasses the entire N-terminal domain exposed to the cytoplasm (Figure 6A). Notably, none of these N-terminal peptides contains the scramblase domain. Confocal imaging of Cox IV staining revealed that all three peptides induced significant mitochondrial morphological changes characterized by a more fragmented mitochondrial network and reduced staining intensity of Cox IV (Figure 6B), suggesting that the mitochondrial targeting sequence is sufficient to initiate mitochondrial stress. However, the longer peptides, particularly N207, induced more pronounced mitochondrial disruption. To assess whether these N-terminal peptides are also sufficient to induce apoptosis, GFP-tagged full-length ATG9B and the N-terminal peptides were again expressed in H1299 cells, followed by Western blot analysis for PARP cleavage. Interestingly, cleavage was observed with peptide expression at levels comparable to full-length ATG9B (Figure 6, C–E). Further, an increase in pyknotic nuclei (Figure 6, F and G), and the presence of floating cells (Figure 6H) were also observed. Taken together, these findings suggest that ATG9B promotes apoptotic cell death through a mechanism dependent, in part, on its mitochondrial localization domain and independent of its lipid scramblase domain (Figure 6I).

As the ATG9A form is without an N-terminal cytoplasmic leader sequence, we next tested if this peptide might be sufficient to direct ATG9A to mitochondria. Three chimeric proteins were constructed by fusing increasing lengths of the ATG9B N-terminus (104, 145, or 207 amino acids) to the N-terminus of ATG9A, generating GFP-ATG9BN104-9A, GFP-ATG9BN145-9A, and GFP-ATG9BN207-9A proteins, respectively (Supplemental Figure S4A). Confocal microscopy revealed that all three chimeric proteins exhibited similar distributions between either the Golgi apparatus (Supplemental Figure S4B) or mitochondria (Supplemental Figure S4C). Subsequent analyses focused on the GFP-ATG9BN145-9A protein and its distribution with either the Golgi (GM130) or mitochondria (Cox IV; Supplemental Figure S4F). As depicted in Supplemental Figure S4, G and H, this chimeric protein localized more prominently to mitochondria than did native ATG9A, although to a lesser extent than ATG9B, indicating that the N-terminal sequence of ATG9B provides a partial recruitment of ATG9A to mitochondria.

To assess whether mitochondrial targeting is sufficient to provide proapoptotic activity to the ATG9A form, the effects of a fusion protein were assessed in which the N-terminal 145 amino acids of ATG9B were fused to the full-length ATG9A (GFP-ATG9BN145-9A), thereby increasing its mitochondrial association (Supplemental Figure S4H). Western blot analysis shows that expression of the chimeric GFP-ATG9BN145-9A protein increases its PARP cleavage compared with ATG9A (Supplemental Figure S4, I and J) while also increasing the percentage of cells with pyknotic nuclei compared with ATG9A (Supplemental Figure S4K). It should be noted that while an increasing trend was observed for both apoptotic responses, neither proved to reach statistical significance.

## DISCUSSION

The goal of this study was to define the localization and function of ATG9B in tumor cells that exhibit a modest expression of this lipid scramblase compared with its paralogue ATG9A. We find that in addition to sharing a localization to the Golgi with ATG9A, ATG9B exhibits a prominent association with mitochondria that appears to be mediated by a 104 amino acid N-terminal targeting domain (Figure 1; Supplemental Figure S1). Over-expression of the tagged forms of ATG9B in tumor cells leads to deformed and fragmented mitochondria that lose their genomic DNA and membrane potential (Figure 2; Supplemental Figures S2 and S3). Despite our efforts to link these structural changes to a mitophagic response, we find only modest evidence for participation in this process (Supplemental Figure S3). Instead, we observe that ATG9B overexpression results in pronounced apoptotic cell death as assessed by a variety of different canonical apoptosis outcomes (Figures 4 and 5). The premise of this mitochondrial-based cell death is reinforced by the dramatic association of tagged ATG9B to the outer mitochondrial surface just before cellular blebbing and death (Figure 5H).

The expression of the ATG9B form in cells and tissues remains somewhat elusive as, unlike the ATG9A homologue, its protein expression in differentiated tissues appears exceptionally modest (https://www.proteinatlas.org/ENSG00000181652-ATG9B/tissue). Further, the expression of ATG9B in the many tumor cell lines we have examined (Figure 1A) is also modest, even in the nontransformed Beas2B cell line that might have been expected to show higher levels. Thus, a direct correlation between low and high expression in neoplastic versus non-neoplastic cells is somewhat tenuous.

Because of the modest expression in cultured cells, we identified the subcellular localization of the ATG9B protein through the expression of tagged constructs. As for its ATG9A counterpart and many other transmembrane proteins, ATG9B exhibits a partial Golgi association in its trafficking pathways, where it may act to transfer membrane to a nascent phagophore (Mattera *et al.*, 2017; De Tito *et al.*, 2020) or perhaps perform a non-autophagic function to sense Golgi stress (Meng *et al.*, 2023). Most noticeable to us was the unique association with the outer mitochondrial membrane in all cells examined. Importantly, this interaction was markedly reduced in cells expressing an ATG9B protein missing the N-terminal 104 residues, a sequence that does not have homology to canonical mitochondrial targeting sequences but can alone target to the mitochondrial surface (Figure 1I). This localization was observed in multiple lung cancer cell lines as well as a hepatoma cell line (Hep3B) that provided a more graphic and confirmatory association. It is unclear to us how this multitransmembrane protein might associate with the outer mitochondrial membrane and whether it does so in a peripheral way or is integrated into the membrane proper via a membrane transporter or vesicle fusion.

Consistent with its mitochondrial association, overexpression of ATG9B induces morphological alterations that include fragmentation, expulsion of the mitochondrial genome into the cytoplasm, and loss of membrane potential. These changes are consistent with the dramatic ultrastructural disruptions showing loss and/or vesiculation of inner cristae. Based on its localization, capacity to participate in autophagy, and substitute for its ATG9A isoform (Chiduza *et al.*, 2024), it seemed likely to us that ATG9B could help drive mitophagy. Despite our extensive efforts, we were unable to record any robust or reproducible mitophagic responses (Supplemental Figure S3). An attempt to promote a mitophagic response was pursued through the expression of both ATG9B and PARKIN together while in the presence of CCCP. Despite these efforts, no meaningful increase in mitophagic outcomes was observed (Supplemental Figure S3, G–K).

What then is the relationship between the role of ATG9B as a putative autophagic protein and an apoptotic inducer? There is a substantial literature, covered in several comprehensive reviews (Tsujimoto and Shimizu, 2005; Booth *et al.*, 2014; Mariño *et al.*, 2014), describing the interplay between these two pathways in what is often termed “autophagy-dependent” or “autophagy-mediated” cell death. Perhaps most relevant is the interaction between the PI3K activator of autophagy BECN1 and key apoptotic proteins such as BCL-2 (Marquez and Xu, 2012), JNK1 (Wei *et al.*, 2008), and even caspase-3 (Zhu *et al.*, 2010). Thus, the specific role played by ATG9B in these two central cellular pathways, and the bias towards autophagy or apoptosis, could change based on how a given tumor cell responds to a specific environment or stimulus.

A role for ATG9B in mitochondria-centric apoptotic cell death is provided by the biochemical assays described in Figures 4 and 5, which are supported by the graphic live cell imaging of tumor cells expressing ATG9B-GFP (Figure 5H). In these sequences, a gradual association with mitochondria is observed as the expression levels of the tagged protein increase over time. By 25–30 h post-transfection, an apparent tipping point is reached when there is a rapid and dramatic recruitment to the organelle surface just before cellular blebbing and death. As ATG9A/B have been shown to possess lipid scramblase activity (Maeda *et al.*, 2020; Chiduza *et al.*, 2024), it is attractive to predict that this pronounced association of ATG9B to mitochondria could alter the outer membrane, leading to cell death. Whether this activity functions to alter the lipid composition of the outer mitochondrial membrane to promote apoptotic cell death in a way similar to that of the human phospholipid scramblase (hPLSCR) family (Sivagnanam *et al.*, 2017) is under study.

Finally, in further support of the premise that an ATG9B mitochondrial-association plays a role in apoptotic cell death, we have expressed N-terminal peptides (N104, N145, and N207) and found that these associate with mitochondria while leading to apoptosis. These findings support a model in which ATG9B promotes mitochondrial apoptosis through its localization, independently of its scramblase domain. How the association of these short peptides with the mitochondrial surface initiates a cell death cascade is unclear. It will be important to define how N-terminal association mediates recruitment of ATG9B to mitochondria and if a specific surface receptor might activate apoptotic cell death. Interestingly, while the localization of an N-terminal ATG9B truncated protein to the mitochondria is markedly reduced, it does appear to induce a cell death response as assessed by PARP cleavage and floating cells (Figure 5, I–K). This was surprising to us and may be a result of several factors that include: a) high enough levels of mitochondrial associated ATG9B to induce a death response, b) there remain undetermined ATG9B domains that help target the truncated protein to the mitochondria, c) other cell death pathways induced by ATG9B that reside off the mitochondria proper, or d) a combination of these factors.

Overall, these findings highlight a putative and novel, nonenzymatic function of ATG9B in promoting mitochondrial apoptosis. Further investigation into this mechanism could provide new therapeutic targets aimed at initiating mitochondrial apoptosis that would attenuate tumorigenesis.

## MATERIAL AND METHODS

### Antibodies and reagents

Primary antibodies: refer to the table of antibody list (Supplemental Table S1). Goat anti-rabbit and goat anti-mouse secondary antibodies conjugated to either Alexa-Fluor-488 or -594 were from Thermo Fisher Scientific (Waltham, MA). CF 405S goat anti-rabbit or goat anti-mouse secondary antibodies were from Biotium (Fremont, CA). HRP-conjugated goat anti-rabbit and goat anti-mouse secondary antibodies used for Western blot analysis were from BioSource International (Camarillo, CA). 1 kb Plus DNA Ladder was from Life Technologies (Grand Island, NY). Miniprep Express Matrix was from MP Biomedicals (Solon, OH). Restriction enzymes were from New England Biolabs (Ipswich, MA). Precision Plus Protein Standards were from Bio-Rad (Hercules, CA), Bafilomycin A1 (Baf-A1) was from Cayman Chemical (Ann Arbor, MI), and all other chemicals and reagents, unless otherwise stated, were from Sigma (St. Louis, MO).

### Plasmid construction

The following primers (Supplemental Table S2) were used to amplify ATG9A WT -pEGFP C1 or ATG9B WT and mutants -pEGFP C1; -pcDNA3.1(Myc) and -pCR3.1(No tag). ATG9A- pEGFP N1 and ATG9B-pEGFP N1 were generous gifts from Dr. Juan S. Bonifacino. The Hsp60-pmCherry N1 was constructed from a human cDNA library. The PCRs were performed using the PCR SuperMix kit (Invitrogen, Carlsbad, CA), and the PCR fragments were ligated into the TA- pCR2.1 vector (Invitrogen, Carlsbad, CA). The ATG9A/9B WT and mutants, Hsp60 inserts from the pCR2.1 constructs were excised by digestion with the corresponding enzymes (Supplemental Table S2) and sub-cloned into the expression vectors pEGFP C1 or N1/pmCherry C1 (Clontech, Palo Alto, CA); pcDNA3.1 and pCR3.1 (Invitrogen).

All plasmids and DNA constructs were verified by restriction enzyme analysis and sequencing (GENEWIZ, South Plainfield, NJ). Sequences were analyzed using DNASTAR analysis software (DNA Star, Madison, WI).

### Cell culture

Human lung epithelial cell line BEAS2B, lung adenocarcinoma cell line HCC4006, and hepatocellular carcinoma Hep3B were purchased from ATCC. All other lung adenocarcinoma cell lines (including H1299, HCC827, H3255, H1975, H2279, H460, and H157 cells) were gifts from Jonathan Kurie (MD Anderson Cancer Center). All lung cell lines were grown in RPMI 1640, while Hep3B cells were maintained in minimum Eagle's medium, both supplemented with 10% fetal bovine serum, 100 U/ml penicillin, and 100 µg/ml streptomycin (Life Technologies, Waltham, MA). All cells were maintained at 37°C in a humidified incubator with 5% CO_2_. Cells were transfected using Lipofectamine 2000 or 3000 (Invitrogen) according to the manufacturer's protocol.

### Immunofluorescence microscopy

For immunofluorescence microscopy, cells cultured on coverslips were rinsed with D-PBS and fixed for 20 min with 2.5% formaldehyde in PIPES buffer (0.1 M Pipes, pH 6.95, 3 mM MgSO_4_, 1 mM EGTA). After rinsing with D-PBS, cells were permeabilized with 0.1% Triton X-100 in D-PBS for 2 min, rinsed in D-PBS, and incubated in blocking buffer (5% normal goat serum and 5% glycerol in D-PBS) for 1 h at 37°C. Cells were incubated in primary antibodies diluted in blocking buffer for 2 h at 37°C, washed repeatedly in D-PBS before incubating in the appropriate fluorescently labeled secondary antibodies diluted in blocking buffer for 1 h at 37°C. Cells were then washed extensively with D-PBS, rinsed with distilled water, and mounted on a glass slide with ProLong Gold antifade mounting media (Invitrogen). Images were acquired using an AxioObserver D.1 epifluorescence microscope (Carl Zeiss, Thornwood, NY) equipped with a 100W mercury lamp, a 63X, 1.4 N.A. oil objective lens, and an Orca II digital camera (Hamamatsu Photonics, Hamamatsu, Japan) or a Zeiss LSM980 confocal microscope with a 63X, 1.4 N.A. oil objective and Zen software. Images were processed using Adobe Photoshop (Adobe Systems Incorporated, San Jose, CA).

### Transmission electron microscopy

For standard TEM, cells on carbon-coated coverslips were rinsed in 37°C Hank's Balanced Salt Solution with Calcium and Magnesium (HBSS) and fixed with 37°C primary fixative (100 mM cacodylate pH 7.4, 60 mM sucrose, 2.5% glutaraldehyde) for 1 h at room temperature, rinsed three times with washing buffer (100 mM cacodylate pH 7.4, 200 mM sucrose) then fixed in the secondary fixative (50 mM cacodylate pH 7.4, 100 mM sucrose, 1% OsO4) for 1 h at room temperature, and rinsed three times in water then fixed in 1% uranyl acetate in water for 1 h at room temperature. Samples were then dehydrated in a graded ethanol series, embedded in Quetol 651 (Ted Pella, Redding, CA), and polymerized in a 65°C oven overnight. After removal from the oven, the coverslip was removed from the bottom of the sample, the block trimmed down to a trapezoid 1 mm wide at the base, and 100 nm thin sections were cut and viewed on a Jeol 1400 transmission electron microscope (Jeol, Tokyo, Japan).

### Protein and membrane extraction

Cells were lysed in ice-cold RIPA lysis buffer (50 mM Tris-HCl pH 8.0, 150 mM NaCl, 1% NP-40, 0.5% sodium deoxycholate, 0.1% SDS) supplemented with Protease Inhibitor Cocktail (P8340, Sigma). Mem-PER Plus Membrane Protein Extraction Kit #89842 (Thermo Scientific) was used for membrane protein isolation according to the manufacturer's protocol.

### Mitochondria isolation and proteinase protection assay

Cellular fractionation was performed using the Thermo Scientific Mitochondria Isolation Kit (Catalogue No. 89874) according to the manufacturer's protocol. To improve the purity of the isolated mitochondria, a low-speed centrifugation step at 3,000 × g, instead of 12,000 × g, was used to separate the mitochondrial and cytosolic fractions. For proteinase protection assays, mitochondria isolated from 1 × 10⁷ cultured cells were resuspended in mitochondrial resuspension buffer and divided into three equal aliquots. Samples were treated with 0, 0.2, or 1 mg/ml trypsin. Reactions were terminated by boiling in Laemmli buffer containing β-mercaptoethanol for 5 min.

### qPCR measurements of mitochondrial DNA

Cytosolic mitochondrial DNA was extracted essentially as previously described (Cao *et al.*, 2020). Briefly, cells were lysed using a mild digitonin-based buffer and fractionated by sequential centrifugation to obtain cytosolic supernatants. Cytosolic DNA was purified using the QIAquick Nucleotide Removal Kit and whole-cell DNA using the QIAamp DNA Mini Kit (Qiagen). Quantitative PCR was performed using an Applied Biosystems system and analyzed using the ΔCt method with cytosolic DNA normalized to the corresponding whole-cell DNA. Human mitochondrial D-loop was amplified using primers 5′-CATCTGGTTCCTACTTCAGGG-3′ and 5′-CCGTGAGTGGTTAATAGGGTG-3′.

### SDS–PAGE and immunoblotting

Equal amounts of protein were resolved on SDS–PAGE gels and transferred to PVDF (Millipore, Burlington, MA) membrane. After blocking with 5% BSA in TBS, the membrane was incubated with primary antibodies at 4°C overnight, washed, and incubated with secondary antibodies for 1 h at room temperature. The signals were detected with SuperSignal West Pico Chemiluminescent Substrate (Thermo Scientific). Secondary horseradish peroxidase-conjugated antibodies (Invitrogen) and HyBlot CL film (LabForce, Muttenz, Switzerland) were used to detect immunoreactive signals. Band densitometry was quantified with VisionWorks LS analysis software (LTF labortechnik, Bodensee, Germany).

### Rhodamine 123 assay

H1299 cells were transfected with either pmCherry C1 or pmCherry C1-ATG9B. 24 h post-transfection, the cells were loaded with 50 nM Rhodamine 123 for 30 min, washed three times with HBSS, and incubated at 37°C for an additional 90 min before imaging. Ten randomly selected images from each group were acquired using both GFP and Texas Red channels. The images were analyzed using Fiji software, where the fluorescence intensity of Rhodamine 123 in each mCherry-positive cell was quantified and graphed. Control mCherry vector expressing cells were pretreated with 20 µM CCCP for 4 h to depolarize the mitochondria before Rhodamine 123 loading, and their fluorescence intensity was set to zero for calibration.

### Annexin V binding assay

H1299 cells were transfected with either a control vector (pmCherry C1) or pmCherry C1-ATG9B. At 24 h post-transfection, both suspended and adherent cells from each group were collected and stained with FITC-Annexin V (Invitrogen) according to the manufacturer's protocol. Flow cytometry was performed using an LSR Fortessa Cell Analyzer (BD Biosciences), and data were analyzed with FlowJo software. Cells were gated for mCherry positivity to exclude non-transfected populations, and the percentage of Annexin V-positive cells was quantified within the mCherry-positive gate.

### mtKeima assay

H1299 cells were transfected with either pcDNA 3.1 control vectors or pcDNA 3.1-ATG9B along with mtKeima vectors. After 24 h, mitophagy was induced by treating the cells for 16 h with 20 µM CCCP. Cells were collected and analyzed by an LSR Fortessa Cell Analyzer (BD Biosciences). Lysosomal mtKeima was measured using dual excitation measurements at 488 nm (pH 7) and 561 nm (pH 4) lasers with 710/50 nm and 610/20 nm detection filters, respectively. For each sample, 100,000 events were collected, and single, mtKeima-positive events were gated based on fluorescence and forward/side scatter properties. Data were analyzed using FlowJo. The proportion of cells undergoing mitophagy was quantified as the percentage of cells with a high 561/488 excitation ratio, indicative of mtKeima localization to the acidic lysosomes.

### Time-lapse live cell microscopy

H1299 cells were transfected with either GFP control vector or GFP-ATG9B along with Hsp60-pmCherry in a 35 mm glass-bottomed imaging dish (Cell E&G, San Diego, CA). Imaging was performed with a Zeiss LSM980 + Airyscan2 confocal microscope (Carl Zeiss, Thornwood, NY) using a 63X oil 1.4 NA objective with stage top incubation set at 37°C and 5% CO_2_. Frames were acquired every 10 min (∼380 frames total). After acquisition, the files were post-processed by the Airyscan module and saved as .czi files. Videos were later exported as .mov files.

### Statistical analysis

For statistical analysis, graphs were generated, and statistical analyses were performed using the two-tailed *t* test using GraphPad Prism software (San Diego, CA). Quantitative data are expressed as means ± SEM. Statistical comparisons were made using Student's *t* test or ANOVA with multiple-comparison tests for the comparisons involving more than two groups, and *p* < 0.05 was considered significant. In experiments where values were expressed as fold changes relative to the control, normalization was performed within each biological replicate, setting the control condition to 1 for each replicate. Statistical significance was then assessed using a one-sample two-tailed *t* test comparing the normalized values to the theoretical mean of 1.

## Supporting information





Supporting Video 1 Movie S1 H1299 cells expressing GFP vector and the mitochondrial marker Hsp60-mCherry for 9 hours prior to imaging exhibit normal mitochondrial dynamics. 1 frame every 10 minutes. Scale bar, 10 μm

Supporting Video 2 Movie S2 ATG9B is recruited to mitochondria rapidly prior to cell death as depicted in the still images from figure 4J. H1299 cells expressing GFP-ATG9B and Hsp60-mCherry for 9 hours prior to imaging 1 frame every 10 minutes. Scale bar, 10 μm.

Supporting Video 3 Movie S3 ATG9B is recruited to mitochondria rapidly prior to cell death as depicted in the still images from figure 4K. H1299 cells expressing GFP-ATG9B and Hsp60-mCherry for 9 hours prior to imaging 1 frame every 10 minutes. Scale bar, 10 μm.

## Abbreviations used:

ATG: autophagy-related protein
BAX: Bcl-2-associated X protein
BCL-2: B-cell lymphoma 2
BECN1: Beclin1
BNIP3: BCL2 interacting protein 3
Cas3: cleaved caspase-3
CCCP: carbonyl cyanide 3-chlorophenolhydrazone
cDNA: complementary DNA
Cox IV: cytochrome c oxidase subunit 4
cyto: cytosolic
DNA: deoxyribonucleic acid
D-PBS: Dulbecco's phosphate-buffered saline
ER: endoplasmic reticulum
FITC: fluorescein isothiocyanate
GFP: green fluorescent protein
GM130: golgin subfamily A member 2
HBSS: Hanks' balanced salt solution
hPLSCR: human phospholipid scramblase
Hsp60: heat shock protein 60
IDC: invasive ductal carcinomas
JNK1: c-Jun N-terminal kinase 1
LAMP1: lysosome-associated membrane protein 1
LC3: microtubule-associated protein 1A/1B-light chain 3
mito: mitochondria
mtDNA: mitochondrial DNA
PARP: poly ADP-ribose polymerase
PCR: polymerase chain reaction
PDI: protein disulfide isomerase
PI3K: phosphatidylinositol 3-kinase
PINK1: PTEN-induced kinase 1
PS: phosphatidylserine
qRT-PCR: quantitative real-time PCR
Rho123: rhodamine 123
RNA: ribonucleic acid
RNAseq: RNA sequencing
ROS: reactive oxygen species
SDSPAGE: sodium dodecyl sulfate-polyacrylamide gel electrophoresis
TFAM: mitochondrial transcription factor A
TOM20: translocase of outer mitochondrial membrane 20
ULK1: Unc-51 like autophagy activating kinase 1
WCL: whole cell lysate.
