# Supplementary material for: ATG9B regulates mitochondrial integrity and apoptotic tumor cell death
Source: Mol Biol Cell. 2026 Apr 10;37(5):ar44. doi: 10.1091/mbc.E25-07-0334 (PMC13322376; doi:10.1091/mbc.E25-07-0334)
Supplement: Supplementary file 1 [file mbc-37-ar44-s001.pdf]

Supplemental Materials

Molecular Biology of the Cell

Cao *et al*.

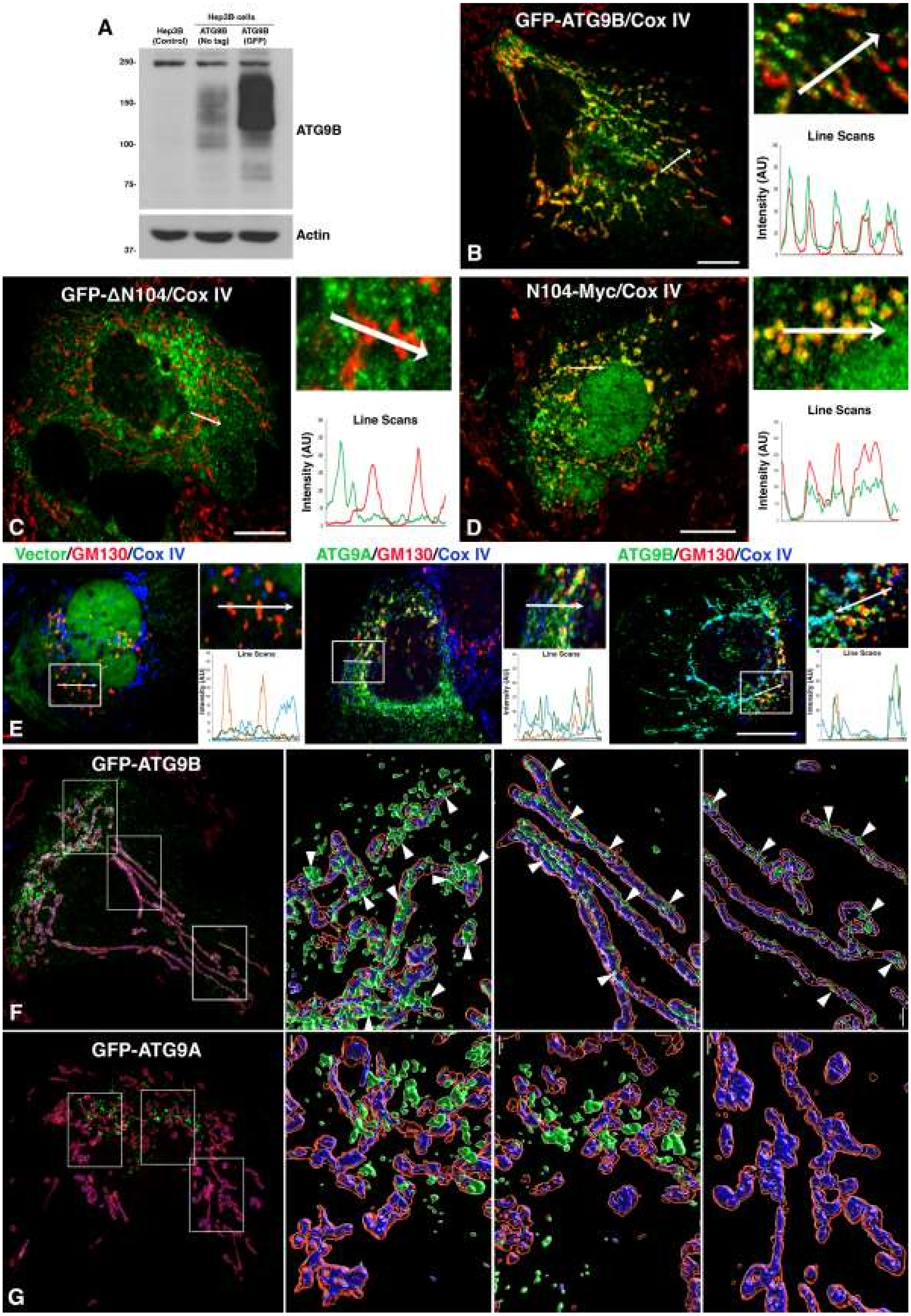

A
Hep3B (Control)
Hep3B cells
ATG9B (No tag)
ATG9B (GFP)
250-
150-
100-
75-
37-
ATG9B
Actin
GFP-ATG9B/Cox IV
Line Scans
Intensity (AU)
B
GFP-ΔN104/Cox IV
Line Scans
Intensity (AU)
C
N104-Myc/Cox IV
Line Scans
Intensity (AU)
D
Vector/GM130/Cox IV
ATG9A/GM130/Cox IV
ATG9B/GM130/Cox IV
E
GFP-ATG9B
F
GFP-ATG9A
G

Figure S1. ATG9B associates with mitochondria in hepatoma cells (Hep3B). (A) Western blot of Hep3B cells overexpressing ATG9B (no epitope tag) or GFP-ATG9B compared to non-transfected cells. (B-D) Confocal images of Hep3B cells overexpressing GFP-ATG9B (B) showing a close alignment along mitochondria [Cox IV (red)], when compared to the GFP-ATG9BΔN104 missing the N-terminus (C). The expression of this N-terminal peptide (N104-Myc) alone exhibits a strong affinity for mitochondria (D). Enlarged views and corresponding line scans are provided in each image highlighting the colocalization of GFP-ATG9B and N104-Myc with Cox IV, as well as the loss of colocalization of GFP-ATG9BΔN104 with Cox IV. (E) Confocal images of Hep3B cells overexpressing GFP vector, GFP-ATG9A, or GFP-ATG9B, co-stained for the Golgi marker GM130 (blue) and Cox IV (red). Enlargements and corresponding line scans along the arrows are shown in the right column of each image depicting the colocalization. (F, G) Fluorescence images showing the localization of (F) GFP-ATG9B (green), or (G) GFP-ATG9A (green) compared to the outer mitochondrial membrane protein TOM20 (translucent red), and the inter-mitochondrial space protein Cox IV (blue). Blow-up boxes show 3D reconstructions from super-resolution Z-stacks. Note the close association of GFP-ATG9B with the mitochondria (arrowheads) that is lacking in the GFP-ATG9A reconstructions. Scale bar, (B, C, E) 10 μm, (F, G) 1 μm.

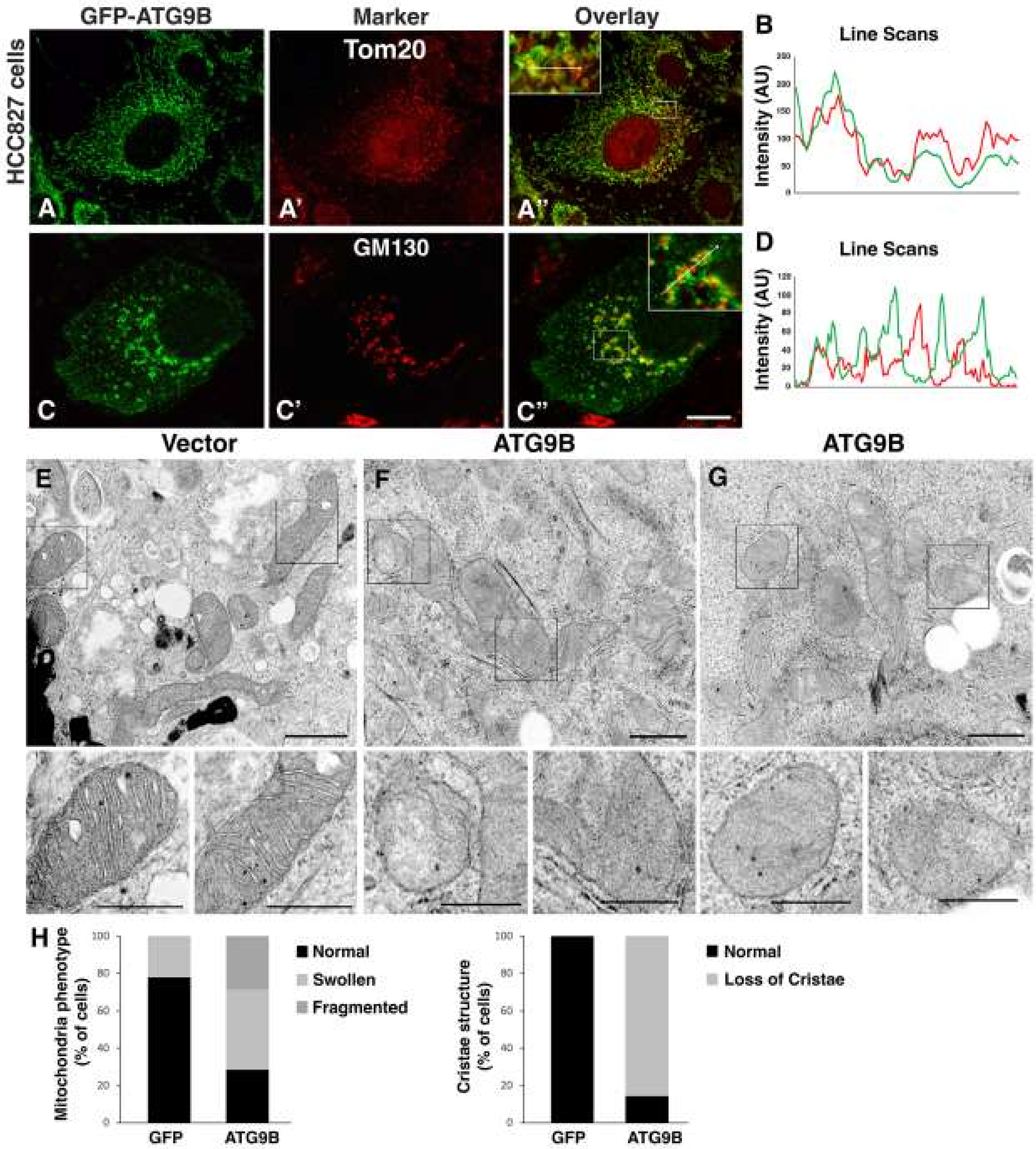

Figure S2. Overexpression of GFP-ATG9B also induces abnormal mitochondrial ultrastructure in a second lung carcinoma cell line HCC827 cells. (A) Confocal images of HCC827 cells overexpressing GFP-ATG9B and immuno-stained for the mitochondrial marker Cox IV (red). (B) Corresponding fluorescence intensity line scans along the arrows in (A") showing marked colocalization between GFP-ATG9B and Cox IV. (C) Confocal images of HCC827 cells overexpressing GFP-ATG9B and immuno-stained for the Golgi marker GM130 (red). (D) Corresponding fluorescence intensity line scans along the arrows in (C") revealing partial

colocalization between GFP-ATG9B and GM130. (E-G) Representative TEM images of HCC827 cells transfected with either the GFP vector (E) or GFP-ATG9B (F, G) for 27 hours. Enlarged views of the boxed regions are shown in the bottom panels of each image, illustrating normal mitochondria in the control cells (E) and mitochondria with a swollen matrix and reduced or disrupted cristae (F, G) in the ATG9B-overexpressing cells. (H) Quantitation of categorized mitochondrial ultrastructural changes observed in the experiment as described in (E-G). Mitochondria were categorized using two complementary criteria: (i) overall mitochondrial morphology, classified as normal, fragmented, or swollen (left panel); and (ii) cristae architecture, classified as normal or showing loss of cristae (right panel). Histogram graphs show the percentage of cells exhibiting each mitochondrial phenotype. Scale bars, (A, C) 10 μm, (E-G) 1 μm, (widefield view), 0.5 μm (enlarged view).

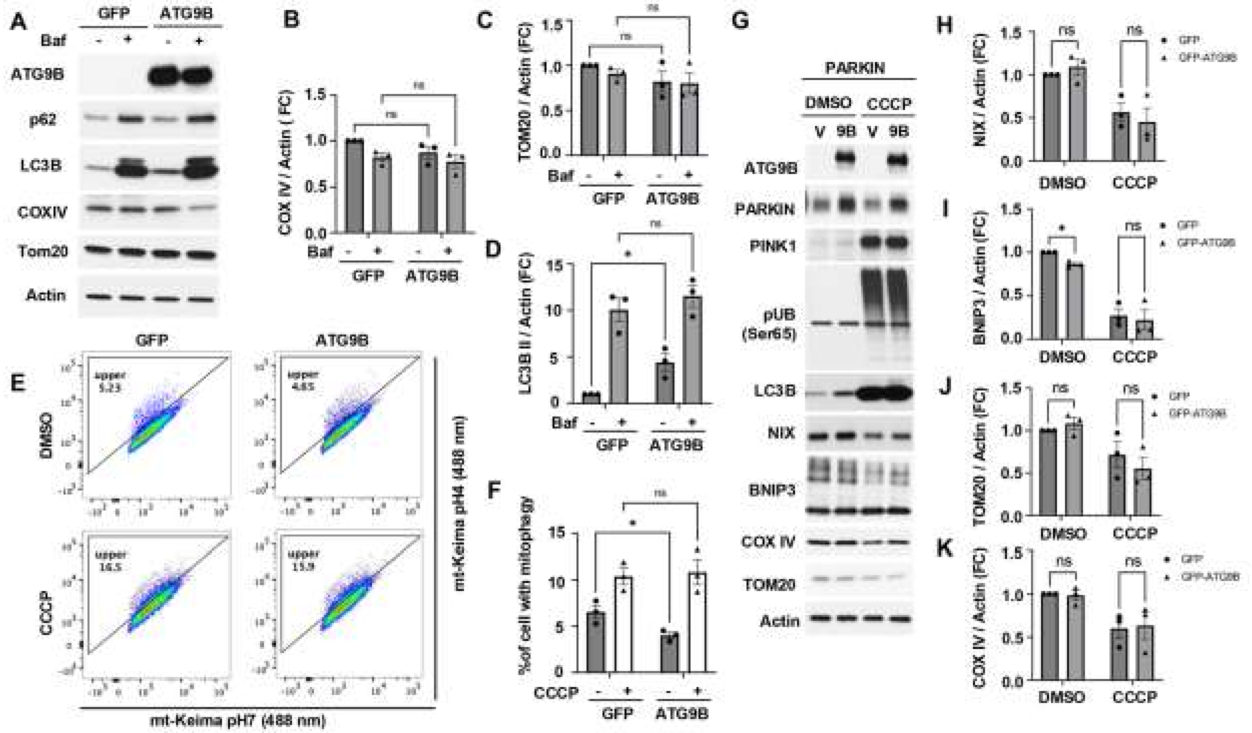

Figure S3. Overexpression of ATG9B promotes only a modest mitophagic response. (A) Western blot analysis of H1299 cells transfected with either the GFP vector or GFP-ATG9B for 24 hours, followed by overnight treatment with or without Bafilomycin A1(1 μM) to inhibit lysosome function. (B–C) Corresponding densitometry analysis of Cox IV (B), TOM20 (C) and LC3B (D) from n=3 independent experiments as described in (A). There are no statistically significant differences in the levels of the mitochondrial markers TOM20 and Cox IV, however, there is a significant increase in LC3B in GFP-ATG9B transfected cells compared to control cells. (E) Representative flow cytometry analysis of mitophagy flux in H1299 cells co-expressing the vital mitophagy probe mtKeima and either the pCR 3.1 vector control or pCR 3.1-ATG9B for 24 hours, followed by overnight treatment with or without the oxidative phosphorylation inhibitor CCCP (20 μM) as a positive mitophagy-inducing control. (F) Bar graph representing the percentage of mitophagic cells measured in experiments as described in (E) (n = 3). ATG9B expression appears

to actually reduce the mitophagic response. (G) Western blot analysis of H1299 cells transfected with mCherry-Parkin and either the GFP control vector (labeled as V) or GFP-ATG9B (labeled as 9B) for 24 hours, followed by overnight treatment with or without CCCP (20 μM). (H–K) Corresponding densitometry analysis of NIX (H), BNIP3(I), Cox IV (J), TOM20 (K), from n=3 independent experiments as described in (F). The analysis shows no statistically significant differences in the levels of the mitochondrial markers. For each Western blot densitometry analysis, target protein levels were quantified as the ratio of target protein to Actin. Within each biological replicate experiment, values were normalized to the GFP control without treatment. The normalized data were then analyzed using a paired *t*-test to assess statistical significance. For the mtKeima assay, statistical analyses were performed using one-way ANOVA with multiple-comparison testing. Bar graphs represent the mean ± SEM, ns: non-significant, * $p < 0.05$.

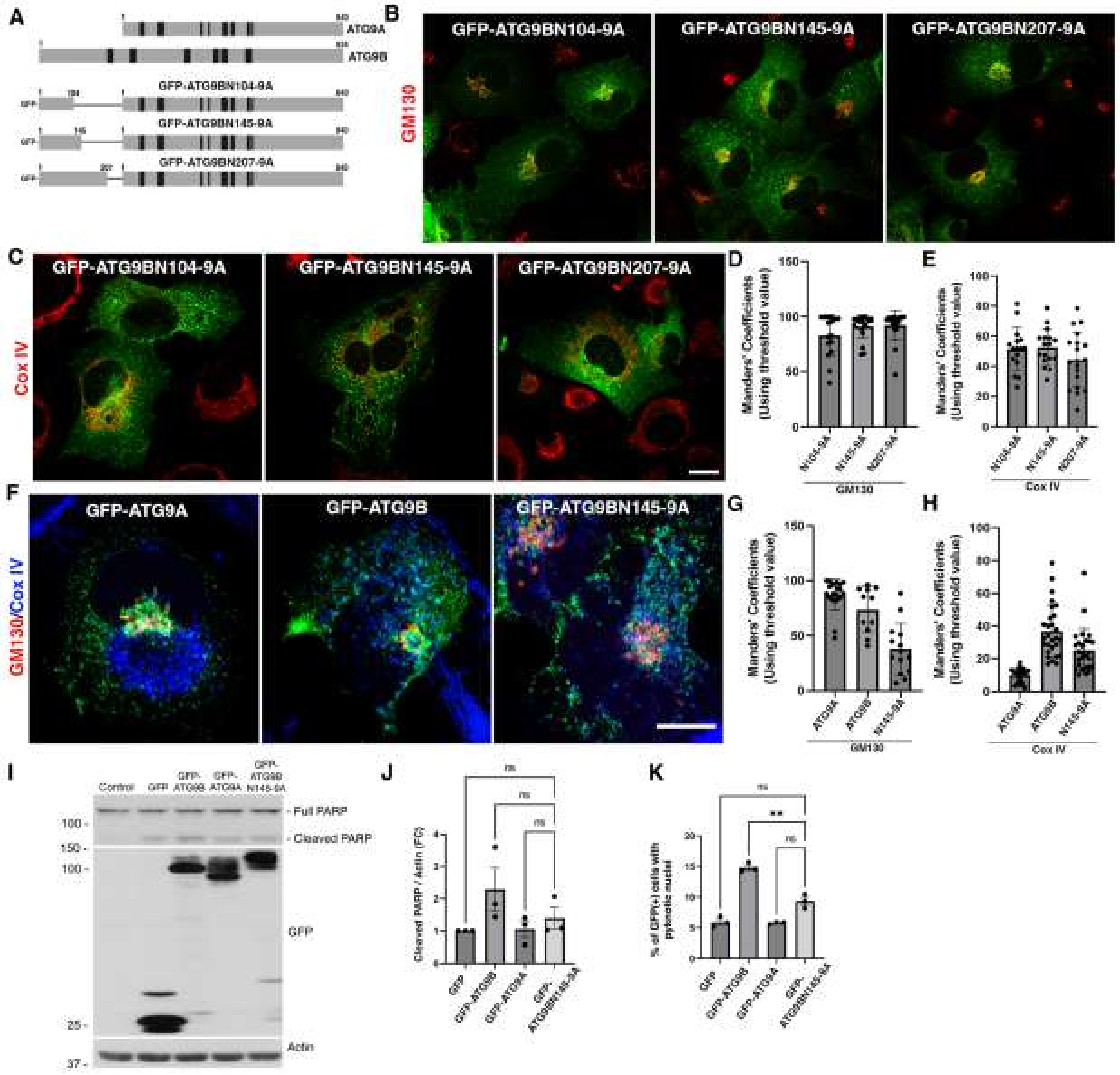

Figure S4. The ATG9B N-terminal region promotes mitochondrial association of ATG9A and supports PARP cleavage. (A) Schematic representation of full-length ATG9A and ATG9B, and GFP-tagged chimeric proteins in which the N-terminal region of ATG9B (amino acids 1-104, 1-145, or 1-207) is fused to ATG9A. (B, C) Confocal microscopy of cells expressing the indicated chimeric proteins and co-stained with (B) the Golgi marker GM130 (red) or (C) the mitochondrial marker Cox IV (red), showing their subcellular localization and colocalization with Golgi and mitochondrial compartments. (D, E) Quantification of colocalization between the chimeric proteins and GM130 (D) or Cox IV (E) using Manders' coefficients. (F) Confocal microscopy of cells expressing GFP-tagged ATG9A, ATG9B, or chimera ATG9BN145-9A and co-stained with GM130 (red) and Cox IV (blue). (G, H) Quantification of colocalization of ATG9A, ATG9B, or chimera ATG9BN145-9A with GM130 (G) or Cox IV (H) using Manders' coefficients. (I-K) Comparative effects of full length or chimeric forms of ATG9B, ATG9A and ATG9BN145-9A on PARP cleavage (I,J) or the formation of pycnotic nuclei (K). Corresponding densitometry analysis of cleaved PARP fold change compared to GFP control from n=3 independent experiments. Bar graph representing the mean percentage of GFP positive cells with pyknotic nuclei post 18 hours of transfection (~900 cells per group per experiment counted, n=3). Bars represent means ± SEM. Statistical analyses were performed using one-way ANOVA followed by multiple comparisons test. ns: non-significant, ** $p<0.01$.

Supplemental Table 1: List of antibodies used in the study.

**Targets for antibodies**	**Brand**	**Catalog Number**
GFP	Abcam	ab290
ATG9A (EPR2450)	Abcam	ab108338
ATG9B	Abcam	ab240897
Bax (6A7)	BD Pharmingen	556467
GM130	BD Transduction lab	610822
Caspase-3	Cell Signaling	9662S
Cleaved Caspase-3 (D175)	Cell Signaling	9661S
Cox IV (3E11)	Cell Signaling	4850S
Cox IV (4D11-B3-E8)	Cell Signaling	11967S
Cyto. C (6H2.B4)	Cell Signaling	12963S
Cyto. C (D18C7)	Cell Signaling	11940
LC3B	Cell Signaling	2775
Myc-tag (9B11)	Cell Signaling	2276

PARP	Cell Signaling	9542
SQSTM1/p62 (D5E2)	Cell Signaling	8025T
TFAM (D5C8)	Cell Signaling	8076S
Tom20 (D8T4N)	Cell Signaling	42406
mtDNA	EMD Millipore	CBL186
Tom20 (2F8.1)	EMD Millipore	MABT166
ATG9B	Invitrogen	PA5-78672
Annexin V-FITC	Invitrogen	A13199
ATG9B	Novus	NBP1-77169
Caspase-3 (L-18)	Santa Cruz	sc-1225
Actin	Sigma	A2066

Supplemental Table 2: List of primers used in the study.

**Plasmid DNA**	**Primers sequence**
pEGFP C1-ATG9A WT5'	AAGCTTTGATGGCGCAGTTTGACACTGAATACCAGCGC
pEGFP C1-ATG9A WT3'	GAATTCTCTATACCTTGTGCACCTGAGGGGGTAGCTCATC
pEGFP C1-ATG9B WT5'	AAGCTTTGATGGTGAGCCGAATGGGCTGGGGCGGAAGAAGAAG
pEGFP C1-ATG9B WT3'	GAATTCTCTCAGTCAGTGCAAGAGGCCCGGTCAGGCTCCTT
pEGFP C1-ATG9B ΔN104 5'	AAGCTTTGATGACACCCGCCTCTGCATCTCCCTCTTGG
ATG9B WT-pCR3.1 (No tag) 5'	AAGCTTTGATGGTGAGCCGAATGGGCTGGGGCGGAAGAAGAAG
ATG9B WT-pCR3.1 (No tag) 3'	GAATTCTCAGTCAGTGCAAGAGGCCCGGTCAGGCTCCTT
ATG9B WT-pcDNA3.1 (Myc) 5'	AAGCTTATGGTGAGCCGAATGGGCTGGGGCGGAAGAAGAAG
ATG9B WT-pcDNA3.1 (Myc)3'	GAATTCCAGTCAGTGCAAGAGGCCCGGTCAGGCTCCTT
ATG9B N104-pcDNA3.1 (Myc) 3'	GAATTCTCTGCAGGTTGAGCCTGTG
ATG9B-N145-pcDNA3.1 (Myc) 3'	GAATTCtAGGGCCTACCCGAAGCCCAGG
ATG9B-N207-pcDNA3.1 (Myc) 3'	GAATTCgGCCATTCCGCTGGTGGTAGC
Hsp60 WT-pmCherry N1 5'	CTCGAGATGCTTCGGTTACCCACAGTCTTTCG
Hsp60 WT-pmCherry N1 3'	AAGCTTATAGAACATGCCACCTCCCATACCAC
